# Odorant Binding Protein C17 Contributes to the Response to *Artemisia vulgaris* Oil in *Tribolium castaneum*

**DOI:** 10.3389/ftox.2021.627470

**Published:** 2021-03-25

**Authors:** Shan-Shan Gao, Rui-Min Li, Shuang Xue, Yuan-Chen Zhang, Yong-Lei Zhang, Jing-Shun Wang, Kun-Peng Zhang

**Affiliations:** ^1^Department of Food and Bioengineering, Innovation and Practice Base for Postdoctors, Anyang Institute of Technology, Anyang, China; ^2^College of Life Sciences, Nanjing Normal University, Nanjing, China

**Keywords:** odorant-binding proteins, *Artemisia vulgaris* essential oil, *Tribolium castaneum*, exogenous toxicants, RNA interference

## Abstract

The red flour beetle, *Tribolium castaneum* (*T. castaneum*), generates great financial losses to the grain storage and food processing industries. Previous studies have shown that essential oil (EO) from *Artemisia vulgaris* (*A. vulgaris*) has strong contact toxicity to larvae of the beetle, and odorant-binding proteins (OBPs) contribute to the defense of larvae against *A. vulgaris*. However, the functions of OBPs in insects defending against plant oil is still not clear. Here, expression of one *OBP* gene, *TcOBPC17*, was significantly induced 12–72 h after EO exposure. Furthermore, compared to the control group, RNA interference (RNAi) against *TcOBPC17* resulted in a higher mortality rate after EO treatment, which suggests that *TcOBPC17* involves in the defense against EO and induces a declining sensitivity to EO. In addition, the tissue expression profile analysis revealed that the expression of *TcOBPC17* was more abundant in the metabolic detoxification organs of the head, fat body, epidermis, and hemolymph than in other larval tissue. The expression profile of developmental stages showed that *TcOBPC17* had a higher level in early and late adult stages than in other developmental stages. Taken together, these results suggest that *TcOBPC17* could participate in the sequestration process of exogenous toxicants in *T. castaneum* larvae.

## Introduction

Insects have sensitive olfactory systems to recognize and receive various odor molecules in their surrounding environments. Through their olfactory system, insects could perform kinds of physiological and reproductive processes (Pelosi et al., 2018; Yan et al., 2020). Odor chemical substances bind with specific proteins in the sensory lymph, which transport them to odorant receptors, eventually triggering a series of stress responses (Pelosi et al., 2006; Leal, 2013). These specific proteins that binding with odor molecules include chemosensory proteins (CSPs) and odorant-binding proteins (OBPs). Combinations between odors and OBPs in insects could initiate a series of odorant recognition and transduction process (Leal et al., 2005; Smith, 2007). The OBPs of insects are highly soluble and globular proteins, which would secrete abundantly in the sensory lymph once odors appear (Sun et al., 2018). They generally locate in the antenna, mouth, and other chemosensory structures, and their role in olfaction has been extensively studied (Gong et al., 2009; Menuz et al., 2014). Recently, several members of these two families with diverse physiological roles were detected in other organs of the insect body (Liu et al., 2016). OBPs are most commonly expressed in the non-sensory organs, such as specific glands, to participate in the release and/or solubility of small molecules, including semiochemicals, hormones and nutrients (Sanchez-Gracia et al., 2009; Xia et al., 2015; Zhang et al., 2015). These studies could reveal more about the role of these proteins in non-olfactory processes.

Furthermore, OBPs have higher levels in antennae than in other apparatuses of adult insects, which suggests they are involved in the odorant recognition of insects (Huang et al., 2018; Zhang X. Q. et al., 2020). The functions of binding affinities of odorant compounds of OBPs have been confirmed using ligand binding experiments *in vitro* in multiple insects, such as *Drosophila melanogaster* (Larter et al., 2016), *Bombyx mori* (Zhou et al., 2009), *Aedes aegypti* (Kim et al., 2017), and *Periplaneta americana* (He et al., 2017). Meanwhile, the exposure of exogenous toxicants results in an evident increase in the expression level of OBPs in multiple insects, and there is a gradual increase in the resistance to toxicants in Coleoptera, Diptera, Lepidoptera, and Neuroptera (Bautista et al., 2015; Li et al., 2015, 2016; Liu et al., 2017; Xiong et al., 2019). These results suggest OBPs take part in the insect resistance to exogenous toxicants. However, the functions of OBPs during the defense against exogenous pesticides in insect is still unclear.

*Tribolium castaneum* (Herbst) (Coleoptera: Tenebrionidae), the red flour beetle, could severely destroy stored grain crops and their processing products (Upadhyay et al., 2018). The deterioration of stored grains in the quality and quantity due to such pest results in billions of dollars in economic losses each year (Li et al., 2011). Currently, chemical fumigants, such as methyl bromide and phosphine, are one of the most effective means to control this pest (Thompson and Reddy, 2016). However, because methyl bromide depletes the ozone layer and phosphine is potentially genotoxic to animals, the employ of these insecticides has been under restriction (Caballero-Gallardo et al., 2011; Kumar et al., 2011; Kalsi and Palli, 2015). Plant EOs, mixture of volatile compounds, extensively serve as bioactive agents. EOs have been increasingly reported as effective insecticides (Li et al., 2010), ovicides (Tunç et al., 2000), antifeedants (Huang et al., 2000), oviposition inhibitors (Ho et al., 1996), and repellents (Ogendo et al., 2008). Plant EOs can replace chemical pesticides as biological agents for the control of *T. castaneum* (Ebadollahi and Jalali Sendi, 2015). Although, most plant EOs contain the neurotoxic thujone, detrimental to human health (Pelkonen et al., 2013), a Chinese traditional plant, *Artemisia vulgaris* does not (Jiang et al., 2019). Therefore, the EOs of *Artemisia vulgaris* can be used as a potential biological insecticide for the control of *T. castaneum*. As an exogenous pesticide, plant EOs usually trigger the defensive response of insects (Wei et al., 2019). In addition, our recent study showed that one OBP gene, *TcOBPC17* (Gene ID: LOC100240681), had a higher expression level in the presence of *A. vulgaris* EO in the late larvae of *T. castaneum* (Gao et al., 2020). Given these results, the present study further dissected the role of TcOBPC17 during the defense response against *A. vulgaris* EO in *T. castaneum* late larvae.

## Materials and Methods

### Insect Rearing

*T. castaneum* was raised at 30°C and 40% humidity in an artificial climate incubator on whole wheat flour containing 5% (w/w) yeast.

### Tissue Samples Collection

Both females and males at different stages of development were collected, including early and late eggs (1 and 3 days old), early and late larvae (1 and 20 days old), early and late pupae (1 and 5 days old), early and late adults (1 and 10 days old). In addition, diverse tissues from the late larvae (the whole larvae, head, epidermis, fat body, gut, and hemolymph) and from the early adults (the whole adult, head, epidermis, fat body, gut, ovary, antenna, testis, and accessory gland) were collected and dissected. The collected tissue samples were immediately frozen in liquid nitrogen and kept at −80°C until RNA isolation.

### RNA Extraction and Quantitative Real-Time PCR

We isolated total RNAs from the collected tissue samples using Trizol reagent (Vazyme, China) according to the manufacturer's instructions. Complementary DNA (cDNA) was synthesized with HiScript Q RT SuperMix for qPCR (Vazyme, China). Quantitative real-time PCR experiments were performed with AceQ SYBR Green Master Mix (Vazyme, China) on the ABI Q6 (Applied Biosystems, USA). Expression of mRNA was normalized to that of the control gene *rps3*. The relative 2^−ΔΔCt^ method was used to calculate the relative expression values (Livak and Schmittgen, 2001). All primers used in this analysis were listed in Supplementary Table 1.

### ORF Sequence Cloning and Phylogenetic Analysis of OBPC17 Genes

To obtain the ORF sequence of the *OBPC17* gene, we used the cDNA templates of late adults to clone the *OBPC17* gene by PCR using its specific primers (Supplementary Table 1). PCR amplification was performed with TransStart FastPfu DNA Polymerase (TransGen, China). Next the purified PCR products were subcloned into the Blunt-Zero Vector via a pEASY-Blunt Cloning Kit (TransGen, China), and positive clones were confirmed by blue-white screening and sequenced by the sequencer (Springen, China). The amino acid sequence of OBPC17 was created and visualized with DNAMAN.

The amino sequences of OBPs reported in other species were downloaded from the NCBI database (https://www.ncbi.nlm.nih.gov/). These sequences were used to construct the phylogenetic tree based on their sequences. The phylogenetic tree was constructed with the neighbor-joining method in MEGA6 with default settings and 1,000 bootstrap replicates. Detailed information on all sequences is provided in Supplementary Figure 2.

### *A. vulgaris* EO Treatment and Bioassay

Late larvae (20 days old) of *T. castaneum* were collected for treatment with *A. vulgaris* EO. The larvae were exposed to 5% *A. vulgaris* EO or acetone (100 μL) for 1 min. Then they were moved on filter papers for 2 min to air dry, subsequently placed into 8 mL glass vials and continued to raise under the standard condition. Then we collected these treated larvae at the indicated time-points (12, 24, 36, 48, 60, 72 h) for subsequent RNA extraction and RT-qPCR experiments.

A total of 30 late larvae of *T. castaneum* in each group were exposed to 100 μL 5% *A. vulgaris* EO for bioassay. The survival status of the treated larvae was observed and recorded at 72 h. If a beetle did not move or respond when touched with tweezers or a brush, it was considered dead. Each bioassay was replicated five times.

### dsRNA Synthesis and Injection

RNA interference was introduced to inhibit expression of *TcOBPC17*. Primers with T7 polymerase recognition promoters were designed to synthesize dsRNA specific to *TcOBPC17* or GFP (Supplementary Table 1). Next 200 ng dsTcOBPC11 was injected into the body cavity of the late larvae by InjectMan 4 (Eppendorf, Germany). Equivalent water or dsGFP were injected and used as the blank or negative control, respectively. After injection with dsRNA, late larvae were reared normally under standard conditions until the follow-up *A. vulgaris* EO bioassay.

## Results

### Identification and Characterization of TcOBPC17 Gene

From the total cDNA from RNA of late larvae, we cloned the open reading frame (ORF) sequence of *TcOBPC17*. The *TcOBPC17* gene contained an ORF sequence of 390 bp, and encoded a protein of 129 amino acids (Supplementary Figure 1). After downloading the OBP sequences reported in other insects, we constructed a phylogenetic tree of the TcOBPC17 protein (Supplementary Figure 2). TcOBPC17 was well-clustered with 19 other OBP proteins, which suggests that TcOBPC17 may perform similar functions as other OBPs.

### Analysis of TcOBPC17 Transcript Abundances in Different Developmental Stages

**Figure 1:**
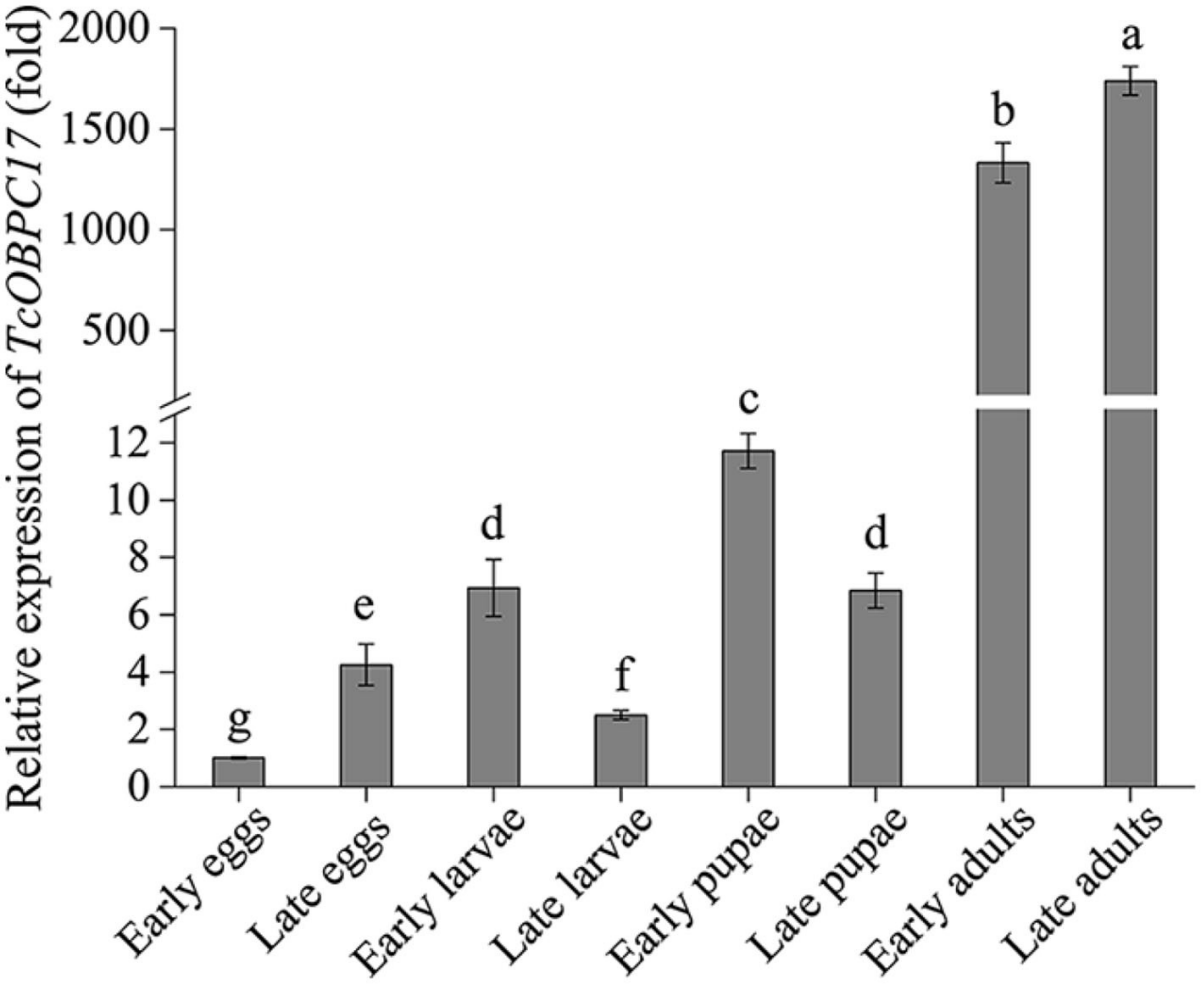
Expression of *TcOBPC17* at eight different developmental stages of *T. castaneum*: early and late eggs (1 and 3 days old), early and late larvae (1 and 20 days old), early and late pupae (1 and 5 days old), early and late adults (1 and 10 days old). The transcript level of *TcOBPC17* in early eggs was used as a calibrator of developmental expression profile. The bars represent standard errors of the means (*n* = 3–5). Fisher's least significant difference (LSD) test was employed to analyze the significant difference between the means, and different letters above the bars represent significant differences in means at *P* < 0.05.

RT-qPCR was performed to investigate the abundance of the *TcOBPC17* transcript at different developmental stages of *T. castaneum* (Figure 1). *TcOBPC17* was expressed ubiquitously throughout all developmental stages of *T. castaneum*, while there were also evident differences in the expression level at different developmental stages. In particular, *TcOBPc17* was highly expressed at the early and late adult stages, and its expression was significantly greater at the adult stages than at other developmental stages.

### Analysis of Tissue Expression Profiles of TcOBPC17

**Figure 2:**
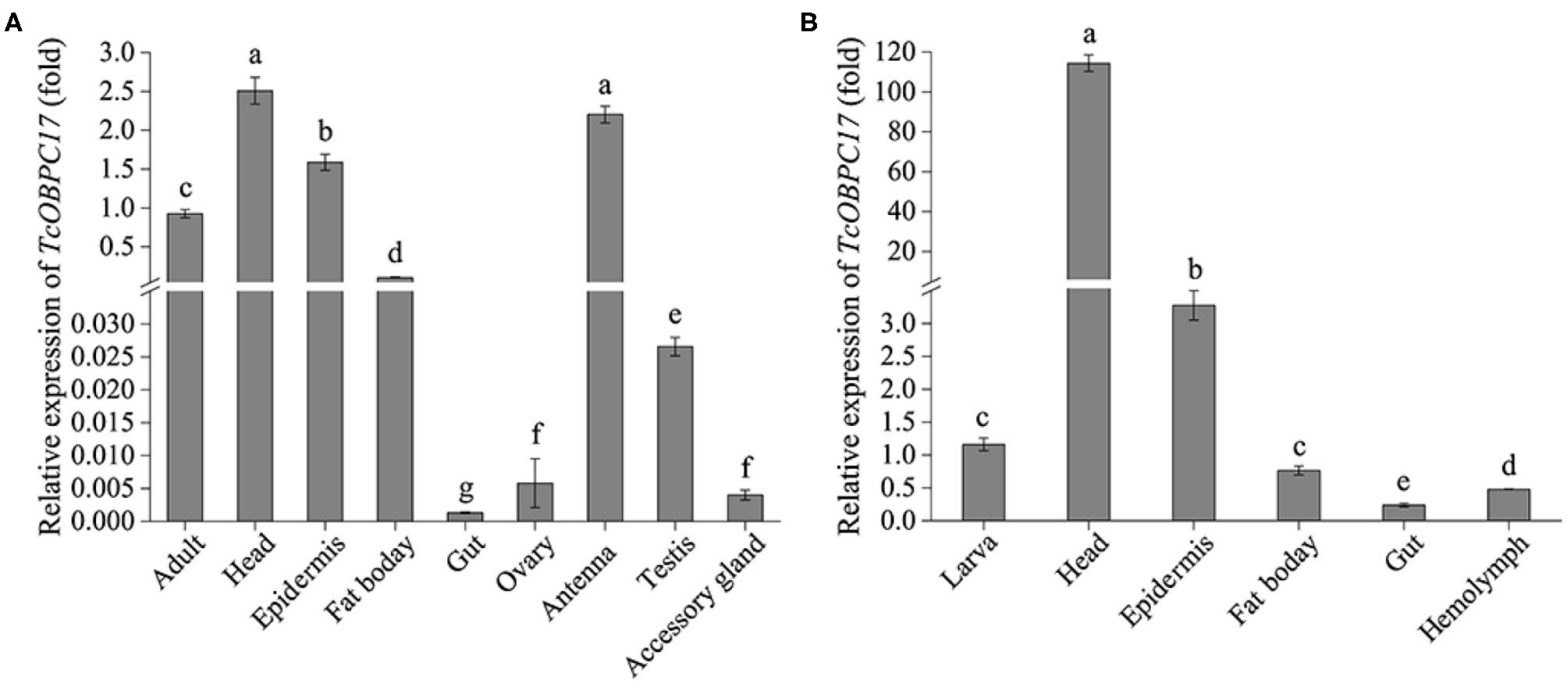
Expression levels of *TcOBPC17* in different tissues of *T. castaneum* late larvae and adults. **(A)** Expression of *TcOBPC17* was measured in different tissues of *T. castaneum* late adults, including the whole adult, head, epidermis, fat body, gut, ovary, antenna, testis, and accessory gland. **(B)** Expression levels of *TcOBPC17* was detected in different tissues of *T. castaneum* late larvae, including the whole larva, head, epidermis, fat body, gut, and hemolymph. The transcript level of *TcOBPC17* in the whole adult or larva served as the calibrator of tissue-specific expression profile. The bars represent standard errors of the means (*n* = 3). After logarithmic processing of *TcOBPC17* levels, significant differences were subjected to Fisher's LSD test. Different letters above the bars represent significant differences in means at *P* < 0.05.

To further explore the tissue expression profiles of *TcOBPC17*, its expression in various tissues from late larvae and late adults was measured. In adult *T. castaneum, TcOBPC17* had the more abundant expression in the head, epidermis, fat body, and antenna; it was expressed much less in the gut, ovary, testis, and accessory gland (Figure 2A). Among these tissues in adults, *TcOBPC17* was most abundant in the head and antenna.

In addition, RT-qPCR was performed to quantify expression of *TcOBPC17* in five tissues of late larvae: the head, epidermis, fat body, gut, and hemolymph. We found that *TcOBPC17* was relatively more abundant in the head and epidermis of late larvae and was extremely abundant in the head, at levels much higher than in the epithelium. Expression was relatively low in the other three tissues compared to the head and epidermis (Figure 2B).

### Dynamic Change in TcOBPC17 Over Time After Treatment With *A. vulgaris* EO

**Figure 3:**
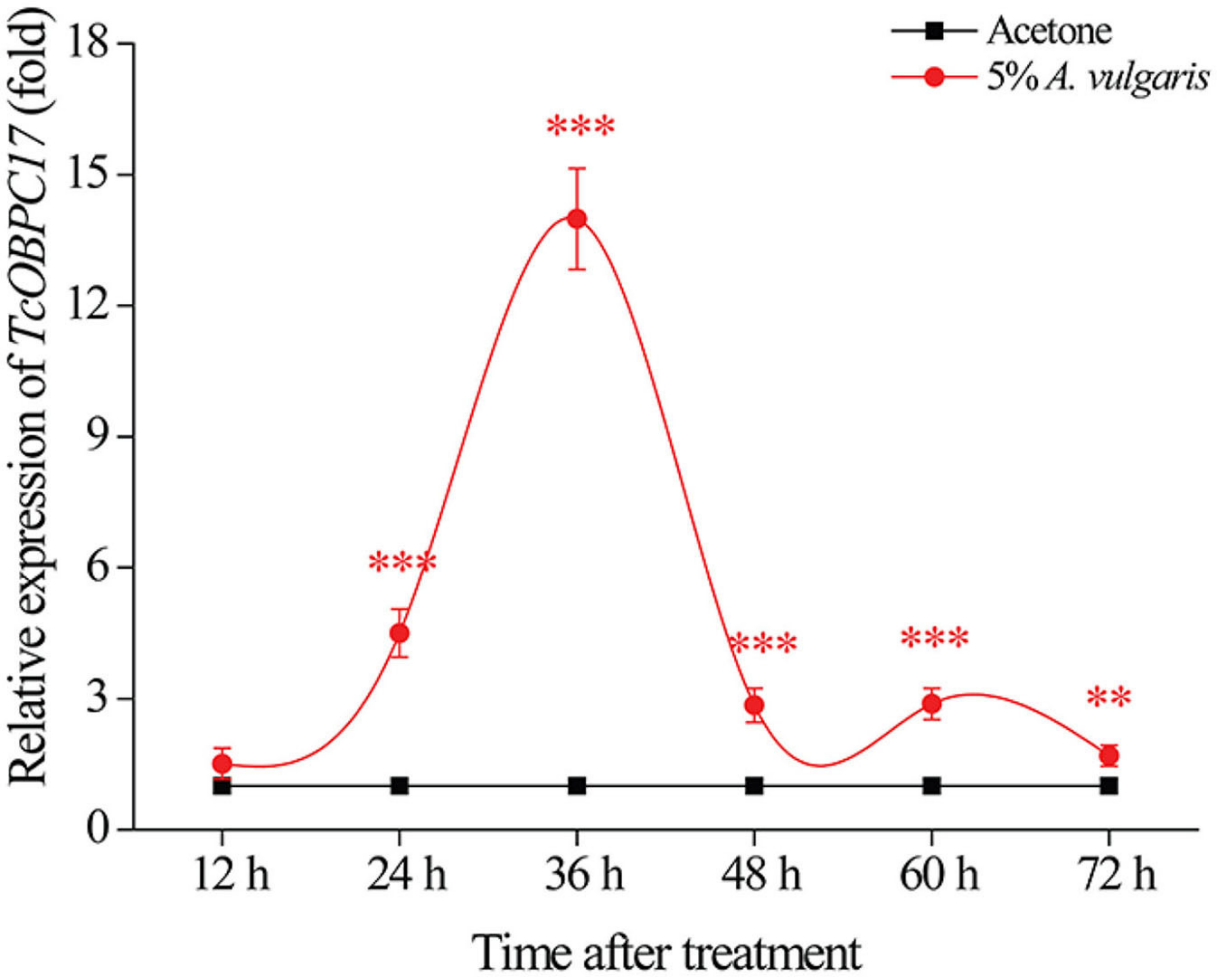
Relative expression level of *TcOBPC17* after exposure to *A. vulgaris* EO or acetone. *TcOBPC17* was measured 12, 24, 36, 48, 60, and 72 h after treatment with 5% *A. vulgaris* EO or acetone. The results are presented as means ± SE (*n* = 3). Student's *t-*test was employed to perform significant difference analysis (**P < 0.01, ***P < 0.001).

To assess the underlying function of *TcOBPC17* during response to *A. vulgaris* EO in *T. castaneum*, we analyzed the temporal expression of *TcOBPC17* in late larvae. We performed RT-qPCR to quantify levels of *TcOBPC17* at six time-points (12, 24, 36, 48, 60, and 72 h) following treatment with 5% *A. vulgaris* EO or acetone (Figure 3). *TcOBPC17* had a significantly higher expression level in larvae treated with *A. vulgaris* EO than larvae treated with acetone from 24 h to 72 h. In addition, after exposure to 5% *A. vulgaris* EO, *TcOBPC17* increased rapidly at 24 h, reached to peak at 36 h, and then reduced gradually to a level approaching that of the control. Taken together, these results indicate that treatment with *A. vulgaris* EO results in fluctuations in the expression of endogenous *TcOBPC17* in *T. castaneum* larvae.

### Functional Analysis of TcOBPC17 Responsed to *A. vulgaris* EO

**Figure 4:**
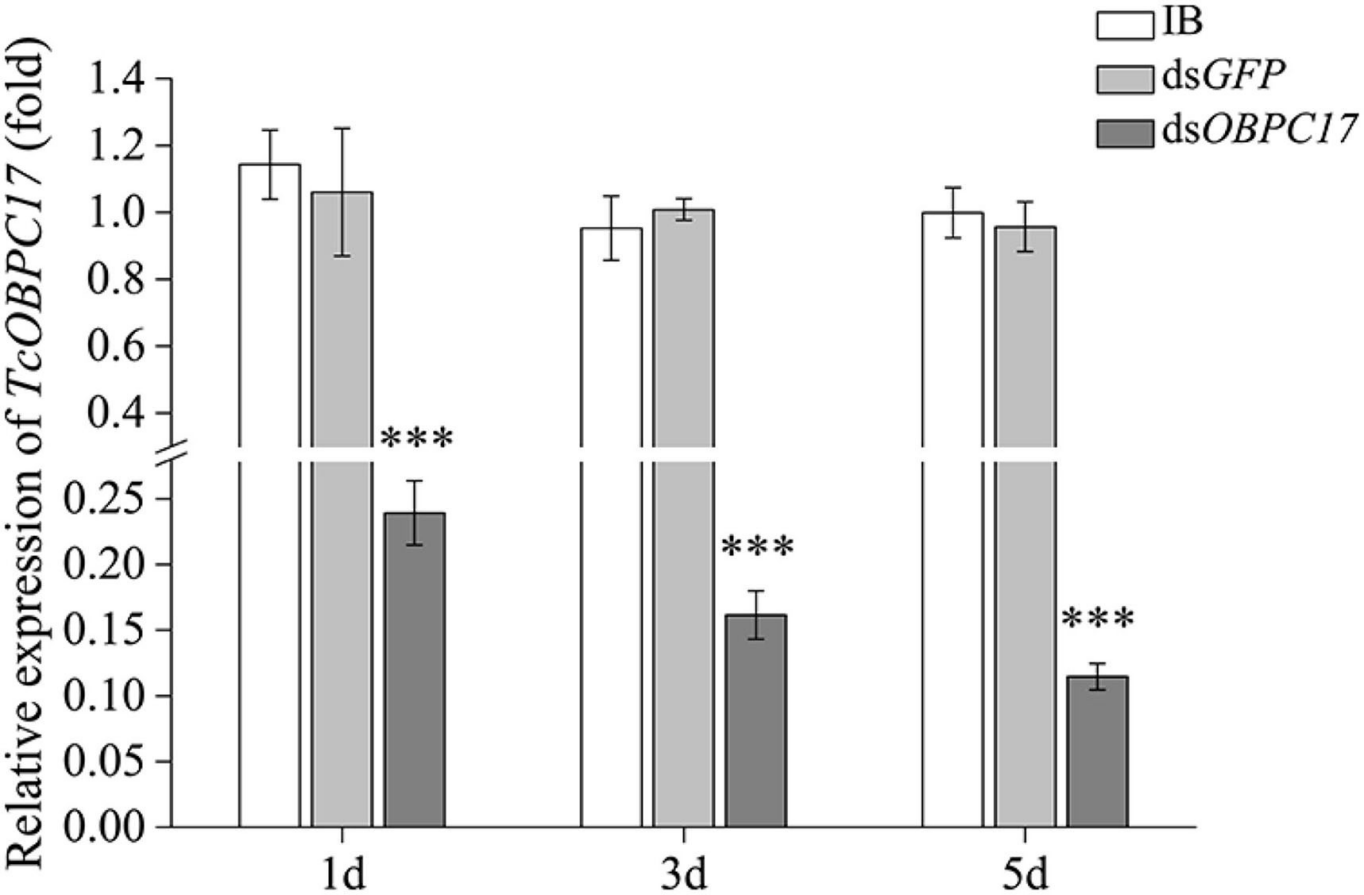
Relative expression level of *TcOBPC17* mRNA in *T. castaneum* 1, 3, and 5 days upon dsOBPC17 injection. The larvae injected with the equal volume of water (IB) or dsGFP served as the controls. The bars represent standard errors of the means (*n* = 3). Fisher's LSD test was employed to analyze the significant differences between the means (***P < 0.001).

**Figure 5:**
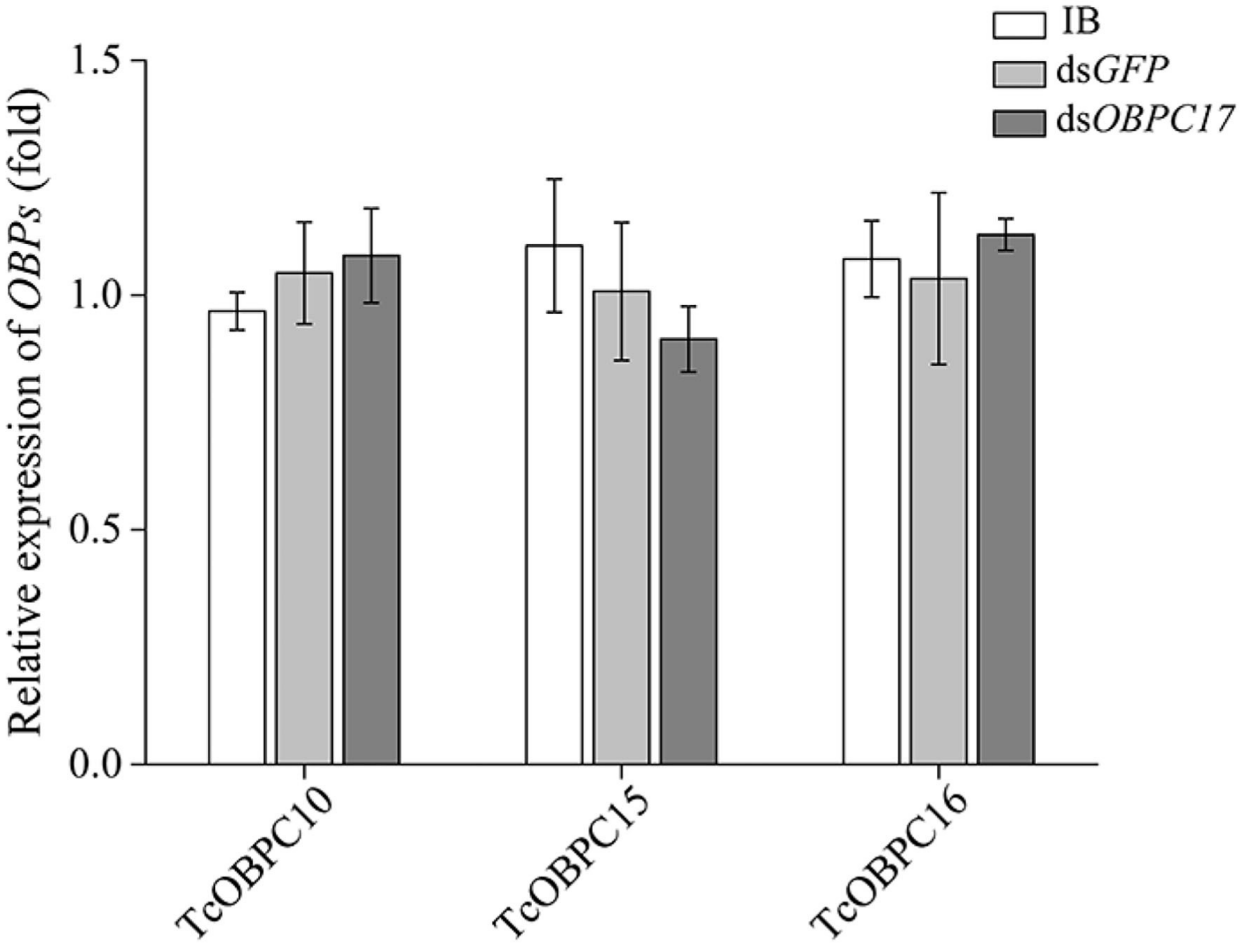
Relative expression of *TcOBPC10, TcOBPC15*, and *TcOBPC16* mRNA in *T. castaneum* 5 days after injection with dsOBPC17. The larvae injected with the equal volume of water (IB) or dsGFP served as the controls. The data are presented as means ± SE (*n* = 3). Fisher's LSD test was employed to analyze the significant difference between the means.

To further evaluate the susceptibility of *TcOBPc17* to *A. vulgaris* EO, we performed an RNAi experiment to silence expression of *TcOBPC17*. We first identified the silence specificity for *TcOBPC17* in *T. castaneum* late larvae by RT-qPCR. *TcOBPC17* was significantly decreased 1, 3, and 5 days after injection with dsTcOBPC17 compared to control water (IB) and dsGFP injection. The fold reduction in *TcOBPC17* was largest at 5 days (Figure 4). Levels of non-target genes, including *TcOBPC10, TcOBPC15*, and *TcOBPC16*, were also measured at 5 days, and they were unchanged (Figure 5). Taken together, these results indicate that RNAi of *TcOBPC17* did not produce off-target effects.

**Figure 6:**
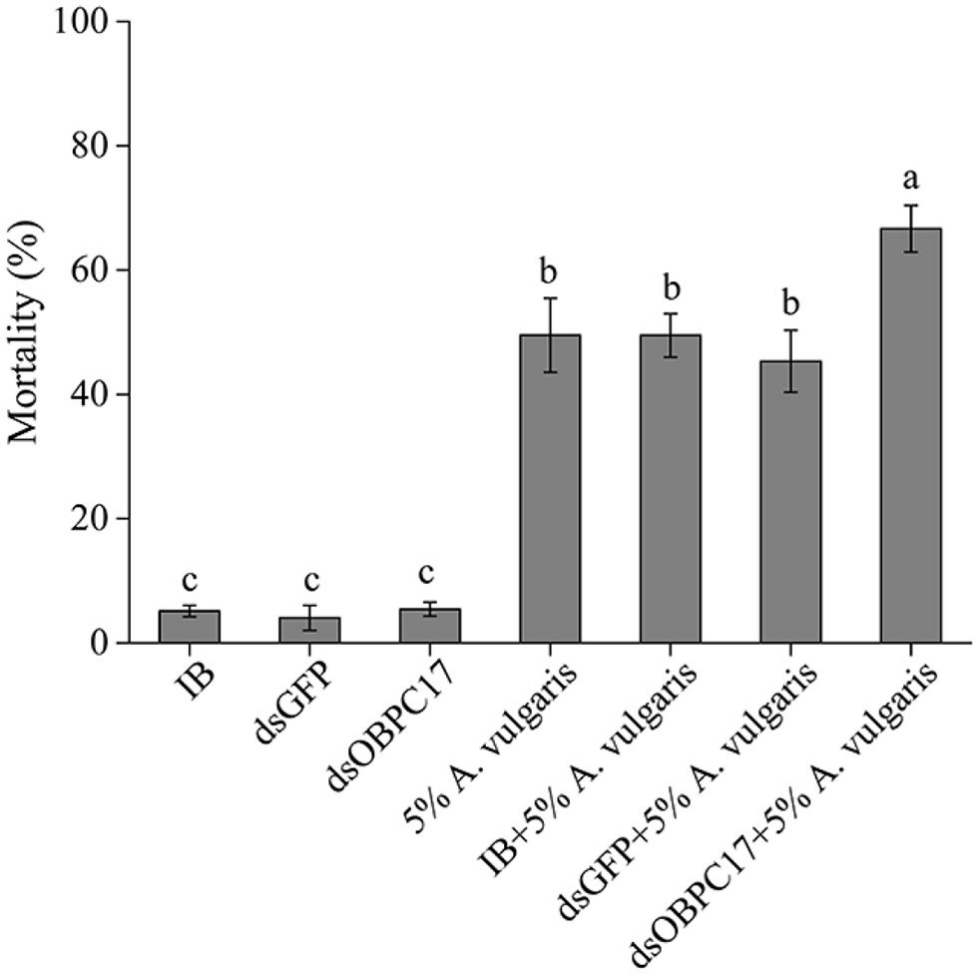
Bioassay of *T. castaneum* larvae exposed to *A. vulgaris* EO after injection with dsOBPC17. The mortality rates of *T. castaneum* larvae were recorded following dsOBPC17 injection with or without exposure to *A. vulgaris* EO (36 h). The larvae injected with the equal volume of water (IB) or dsGFP served as the controls. The bars indicate the standard errors of three to seven biological replicates. The statistical significance of the mortality rate was analyzed with Fisher's LSD test. Different letters above the error bars represent significant differences among treatments at *P* < 0.05.

Dip bioassays of *A. vulgaris* EO were performed 5 days after late larvae were injected with or without dsRNA. Five days after injection with water (IB), dsGFP, or dsOBPC17, the survival rate of *T. castaneum* larvae was 94.85 ± 0.92%, 95.96 ± 2.02%, 94.55 ± 1.12%, respectively; there were no significant differences among treatments. The accumulated mortality of late larvae had obvious rises in the control larvae with water, and also dsGFP, and dsOBPC17 injection groups after exposure to *A. vulgaris* EO. Meanwhile, compared to the water and dsGFP control groups, the mortality of larvae injected with dsOBPC17 significantly increased (Figure 6). These results support the conclusion that increased mortality of late larvae after *A. vulgaris* EO exposure was principally due to silencing of *TcOBPC17* genes. Overall, TcOBPC17 plays a crucial role during response to *A. vulgaris* EO in *T. castaneum* larvae.

## Discussion

Insects prevailingly take advantage of the olfactory system to detect and recognize kinds of odorants from the external environment (Hansson and Stensmyr, 2011). Various sensory sensilla in the olfactory system play essential roles in locating shelter, food, mates, and threats (Kaissling, 2001; Hallem and Carlson, 2006). OBPs, which function as carriers of external odorants, are highly expressed in the antennae in sensillar lymph, so they play vital roles in the olfaction of insect. However, few OBPs have predominant or specific expression in olfaction-related organs, while most are widely expressed in various sensory and non-sensory tissues, which suggests that these OBPs may share other roles (Graham et al., 2003). In this study, to understand the potential function of OBPC17 in *T. castaneum*, we characterized the tissue expression profile by RT-qPCR (Figure 1). We found that *TcOBPC17* was highly expressed in the antenna of late adults, which is in line with the facts that OBPs play key roles in chemosensory process and the antennae are the major chemosensory organs. Moreover, *TcOBPC17* was expressed more highly in the head of late adults and larvae than in other tissues tested. The mouthpart appendages located in the insect head with a variety of chemosensory sensilla, are essential for the feeding of insects (Zhou, 2010).

To better understand the potential function of OBPC17 in *T. castaneum*, we analyzed the transcript abundance of *TcOBPC17* at different developmental stages (Figure 2). OBPs have expressions in both the larval and adult stages in insects (Xue et al., 2016; Zhang et al., 2018). Consistent with this, we found that *TcOBPC1*7 was transcribed throughout all developmental stages, from egg to adult, which suggests that it involves in the multiple physiological activities. We also found *TcOBPC17* had an upregulated expression prominently at the late adult stage of *T. castaneum*. This is similar to the expression of many other OBPs, such as *CcapOBP83a-2* from *Bactrocera capitata* (Siciliano et al., 2014), *OBP1, OBP3, OBP8, OBP11*, and *OBP24* from *Chilo suppressalis* (Yang et al., 2016), and *BdOBP56d, BdOBP99a, BdOBP99c*, and *BdOBP19* from *Bactrocera dorsalis* (Zhang et al., 2018). These similar phenomena are explained to possibly associate with the acquisition of heterosexual behavior and oviposition signals of adult insects (Zhang et al., 2018). We speculate that *TcOBPC17* is most likely involved in mating behavior and not oviposition given the living habits of *T. castaneum*. In addition, *TcOBPC17* was highly expressed in late larvae, suggesting that it may be participated in the development process of *T. castaneum*. The tissue and developmental expression of *TcOBPC17* were very similar to those of *TcOBPC11* in our previous studies (Zhang Y. C. et al., 2020). Further studies are required to confirm whether they have similar functions.

*T. castaneum* is a common pest in stored wheat flour and other foods. Green biological control of *T. castaneum* is particularly important for food safety and quality. In recent years, more and more plant volatiles have been used in pest control to avoid environmental pollution (Ebadollahi and Jalali Sendi, 2015). For example, EO of Artemisia plants displays fumigation, contact, and repellent toxicity to the larvae or adults of *T. castaneum* (Abou-Taleb et al., 2016; Hu et al., 2019). Our previous study also showed strong contact toxicity of *A. vulgaris* EO to late larvae of *T. castaneum* (Gao et al., 2020; Zhang Y. C. et al., 2020). In this study, we examined whether OBPs play roles in the response to EO in *T. castaneum*. Our results showed that, after treatment with *A. vulgaris* EO, expression of *TcOBPC17* was significantly upregulated (Figure 3). Moreover, the introduction of dsOBPC17 to reduce *TcOBPC17* (Figure 4) enhanced the sensitivity of *T. castaneum* to *A. vulgaris* EO and resulted in a poor survival outcome (Figure 6), which proves that *TcOBPC17* plays a key role in the defense against toxic substances in the red flour beetle.

## Conclusions

Our expression profiles by tissue and developmental stage, as well as our RNAi experiments, show that the potential function of TcOBPC17 in the olfactory system of *T. castaneum* and reveal that *TcOBPC17* is indispensable in resistance to exogenous toxic substances. As a whole, our results not only provide essential evidence of the effects of plant volatiles on pest insects' olfactory systems but also significant suggestions for environmentally friendly pest management in the future. Additionally, it is difficult and expensive to obtain sufficient quantities of *A. vulgaris* EO to make pesticides. Therefore, we plan to identify the main/active components of the *A. vulgaris* EO using mass spectrometry. We will then artificially synthesize these monomers and assess their insecticidal activity analysis to identify the main active components in the EO. These components will then be used to make insecticides. This approach will reduce the cost of insecticide production and eliminate the need to obtain large quantities of EO.

## Data Availability Statement

The original contributions presented in the study are included in the article/Supplementary Material, further inquiries can be directed to the corresponding author/s.

## Author Contributions

S-SG and R-ML: conceptualization, methodology, writing—original draft preparation, and writing—review and editing. Y-CZ: software. SX, Y-LZ, and R-ML: validation. R-ML: formal analysis. S-SG and SX: investigation. J-SW: resources. S-SG: data curation and visualization. K-PZ: supervision, project administration, and funding acquisition. All authors contributed to the article and approved the submitted version.

## Conflict of Interest

The authors declare that the research was conducted in the absence of any commercial or financial relationships that could be construed as a potential conflict of interest.

## Supplementary Material

Supplementary Figure 1 ORF and amino acid sequences of the TcOBPC17 gene. The ORF sequence of the TcOBPC17 gene was identified with DNAMAN. The corresponding amino acids are shown below the ORF sequence.

Supplementary Figure 2 The phylogenetic tree of TcOBPC17 and other OBP sequences reported in other insects. The phylogenetic tree was constructed with the neighbor-joining method using MEGA 6.1 software. Bootstrap values (2,000 replicates) are displayed next to the branches of the phylogram. The GenBank accession numbers, full gene names, and scientific names of these insects are exhibited behind the branches of the phylogram.

Supplementary Table 1 Primers used in this study.

## Abbreviations

*T. castaneum*: *Tribolium castaneum*
EO: essential oil
*A. vulgaris*: *Artemisia vulgaris*
OBPs: odorant-binding proteins
RNAi: RNA interference
CSPs: chemosensory proteins
cDNA: complementary DNA.
