# Supplementary figures and images for: Odorant Binding Protein C17 Contributes to the Response to Artemisia vulgaris Oil in Tribolium castaneum
Source: Front Toxicol. 2021 Mar 25;3:627470. doi: 10.3389/ftox.2021.627470 (PMC8979489; doi:10.3389/ftox.2021.627470)

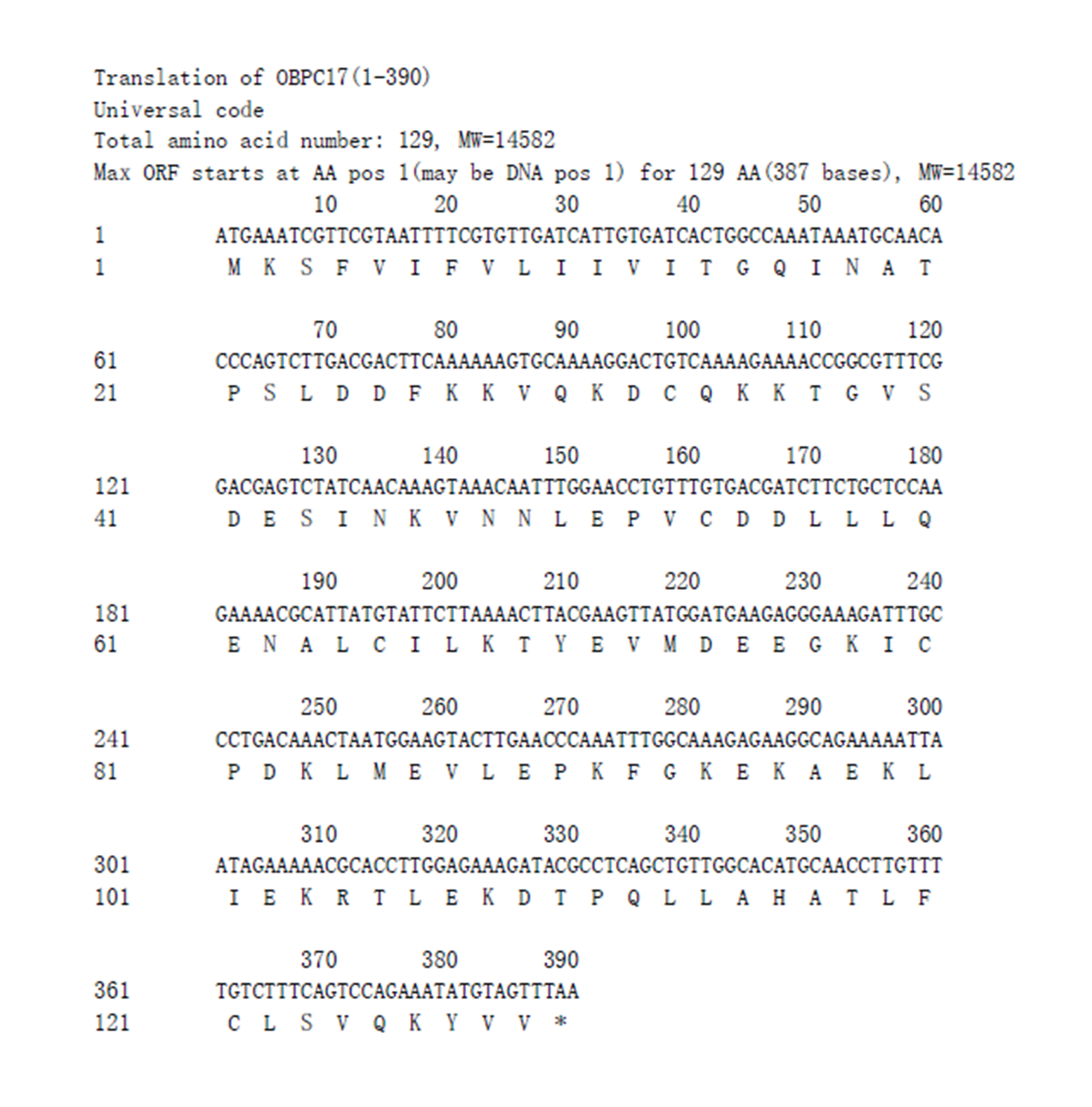

Supplement: Supplementary Figure 1 — ORF and amino acid sequences of the TcOBPC17 gene. The ORF sequence of the TcOBPC17 gene was identified with DNAMAN. The corresponding amino acids are shown below the ORF sequence. [file Image_1.PNG]

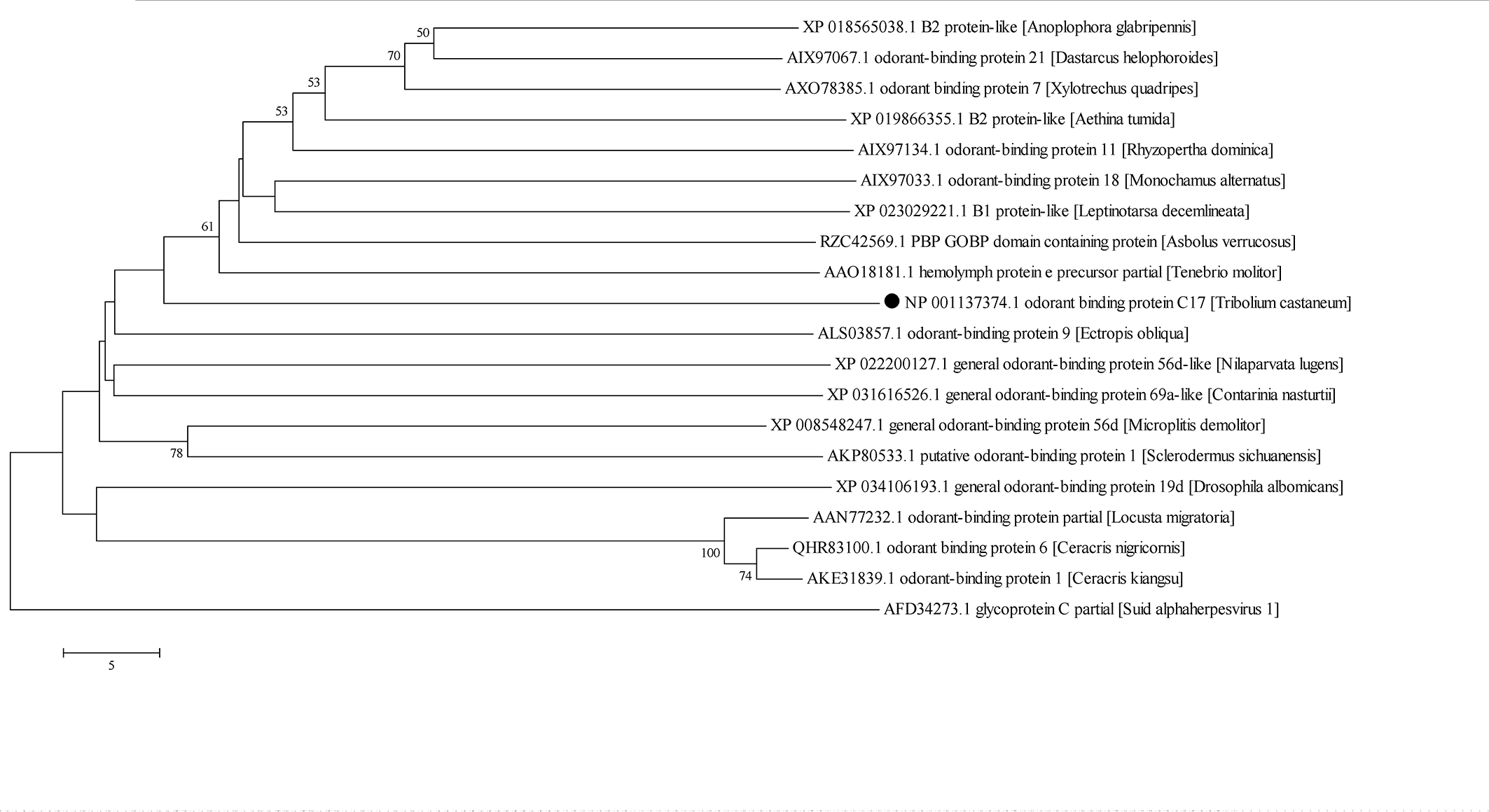

Supplement: Supplementary Figure 2 — The phylogenetic tree of TcOBPC17 and other OBP sequences reported in other insects. The phylogenetic tree was constructed with the neighbor-joining method using MEGA 6.1 software. Bootstrap values (2,000 replicates) are displayed next to the branches of the phylogram. The GenBank accession numbers, full gene names, and scientific names of these insects are exhibited behind the branches of the phylogram. [file Image_2.TIF]
