# Supplementary material for: Odorant Binding Protein C17 Contributes to the Response to Artemisia vulgaris Oil in Tribolium castaneum
Source: Front Toxicol. 2021 Mar 25;3:627470. doi: 10.3389/ftox.2021.627470 (PMC8979489; doi:10.3389/ftox.2021.627470)
Supplement: Supplementary Table 1 — Primers used in this study. [file Table_1.DOCX]

| gene | Sequence(5’-3’) | Primer length (bp) | Product length (bp) | Utility |
| --- | --- | --- | --- | --- |
| *OBPC17-F* | GGCGTTTCGGACGAGTCTATC | 21 | 121 | qRT-PCR |
| *OBPC17-R* | TTCCCTCTTCATCCATAACTTCG | 23 |  |  |
| *OBPC17-F* | TAATACGACTCACTATAGGGTACGAAGTTATGGATGAAGAGGGA | 44 | 166 | RNAi |
| *OBPC17-R* | TAATACGACTCACTATAGGGGGACTGAAAGACAAAACAAGGTTG | 44 |  |  |
| *OBPC17-FF* | ATGAAATCGTTCGTAATTTT | 20 | 400 | Gene cloning |
| *OBPC17-FR* | TTAAACTACATATTTCTGGACTG | 23 | 400 |  |
| *OBPC16-F* | TCTCCACTTTAGTCGCAATCCTT | 23 | 155 | qRT-PCR |
| *OBPC16-R* | CCCCGTTTCGCTTAGTTCTT | 20 |  |  |
| *OBPC15-F* | AGCTGAGGGTCGTAATTTGTCG | 22 | 133 | qRT-PCR |
| *OBPC15-R* | GATTTTGCCGACGTTGTCCT | 20 |  |  |
| *OBPC10-F* | AGATTTTATTCGTTAAAATGGCAAT | 25 | 127 | qRT-PCR |
| *OBPC10-R* | TTTGGCTACGTCCTGAGGGTC | 21 |  |  |
| *GFP*-*F* | TAATACGACTCACTATAGGGCGATGCCACCT | 31 | 500 | RNAi |
| *GFP-R* | TAATACGACTCACTATAGGGTGTCGCCCTCG | 31 |  |  |
| *RPS3*-*F* | TCAAATTGATCGGAGGTTTG | 20 | 260 | qRT-PCR |
| *RPS3*-*R* | GTCCCACGGCAACATAATCT | 20 |  |  |

**Table S1.** Primers used in this study

Note: F represents forward primers; R represents reverse primers. The letters with underline are the T7 promoters for dsRNA synthesis
